# Food Waste and Lunar Phases: Evidence from a Randomized Controlled Trial

**DOI:** 10.3390/foods13050705

**Published:** 2024-02-26

**Authors:** Peng Shan, Lei Zhang, Shiyan Jiang

**Affiliations:** School of Economics and Management, China University of Mining and Technology, Xuzhou 221116, China; shanpeng1996@126.com (P.S.); jsy19951007@163.com (S.J.)

**Keywords:** food waste, new moon phase, full moon phase, randomized controlled trials, propensity score matching

## Abstract

To examine a potential correlation between food waste and lunar phases, we have devised a randomized controlled trial. The experiment spanned from 31 March to 10 July 2022, during which we employed the direct weighing method to collect 1903 valid data points on food waste. Utilizing propensity score matching, we meticulously controlled for various factors, including dining dates, the number of diners, dining times, spending levels, and store activities. The study revealed a close relationship between lunar phases and food waste. During the new moon phase, there was an increase in both orders and waste generated by consumers. Specifically, individuals, on average, squandered an additional 6.27% of animal protein (0.79 g), 24.5% of plant protein (1.26 g), 60.95% of starchy foods (3.86 g), and 61.09% of vegetables (5.12 g), resulting in an aggregate food waste of 32.14% (10.79 g). Conversely, during the full moon phase, consumers decreased their orders and subsequently decreased food waste. On average, individuals wasted 44.65% less animal protein (5.76 g), 43.36% less plant protein (2.5 g), 85.39% less seafood (0.73 g), and 8.43% less vegetables (0.93 g), resulting in a 20.52% (7.81 g) reduction in food waste. Furthermore, we validated our conclusions through various validation methods, including model replacement, to ensure robustness and reliability.

## 1. Introduction

According to the “Global Food Crisis Report 2023” by the Food and Agriculture Organization of the United Nations (FAO), the global population facing severe food insecurity increased to 258 million in 2022. Simultaneously, the issue of food waste is alarming, with approximately 31% of food being lost or wasted globally each year. Food losses primarily occur during harvesting [1], storage [2], processing [3], and other stages. The main reasons for these losses are mainly limited by technological levels. The development of technology raises hope that food losses will continue to decrease. Food waste refers to the edible portion that is not consumed. It is influenced by various factors such as economic income [4] and personal character [5,6]. Reducing food waste is a challenging task as it is primarily caused by subjective factors rather than technological limitations.

Food waste, being a global problem, has gained widespread attention [7]. Scholars primarily focus on investigating the causes of food waste and how to reduce it. Firstly, there are numerous factors influencing food waste. Some scholars have researched the impact of macro-factors such as religious belief [8] and social custom [9] on food waste. Others have studied the influence of micro-factors such as dietary habits [10], economic income [4], and personal character [5,6]. Some scholars have also examined food waste in specific dining environments, such as households [11,12,13,14], schools [8,15,16,17], and the catering industry [18,19,20]. Secondly, research on reducing food waste commonly uses the randomized controlled trial (RCT) paradigm, which establishes experimental and control groups. Various intervention measures such as informational intervention [21] and economic intervention [22] are implemented to study the effects of different types of interventions in reducing waste. The objective is to identify effectiveness and easily scalable intervention measures.

The means used to evaluate food waste commonly include the diary method [23,24], archaeological [25], waste composition analysis (WCA) [26], and direct weighing methods [27,28]. The diary method often compromises data accuracy due to errors or omissions made by participants. The archaeological method is limited in its ability to quantify undiscarded food waste, leading to a potential underestimation of the true quantity. Similarly, criticism is directed towards WCA for its diminished credibility. In contrast, the direct weighing method entails survey participants physically measuring the weight of each food waste type, resulting in enhanced data accuracy and credibility. The methods commonly employed to investigate food waste include questionnaire surveys and the RCT. While questionnaires to some extent reflect individuals’ attitudes towards food waste, it should be noted that food waste is a complex systemic issue influenced not only by individuals’ attitudes but also by various factors. As a result, inconsistencies may arise between attitudes and behaviors. The issue of food waste, which is essentially an individual’s behavior, can be better understood through RCTs. This method can penetrate consciousness and unveil the distinct impact of specific factors on food waste.

It is important to note that the main culprits of food waste are individuals who, apart from being influenced by cultural factors and dining environments, may also be affected by natural cyclic rhythms. For instance, Bashir Adelodun (2021) [29] discovered a significant seasonal cycle in food waste. However, no scholars have yet investigated the impact of lunar phases, a particularly unique cycle, on food waste. Based on extant research, lunar phases may potentially exert influence across multiple domains on human beings. Full moons have been associated with increased traffic accidents [30], crime rates [31], gastrointestinal bleeding risks [32], and disrupted sleep quality [33]. After collecting 1903 instances of food waste data, we found a close correlation between lunar phases and food waste. Specifically, during the new moon, consumers tend to waste more food. Conversely, during the full moon, consumers reduce their food waste compared to other times. However, the reasons behind the fluctuation in food waste according to lunar phases remain unclear. We note that the use of lunar calendars, such as the Islamic and Chinese lunar calendars, in regions that follow the lunar cycle may contribute to cyclical patterns of food waste.

To further explore the relationship between lunar phases and food waste, we conducted a randomized trial spanning from 31 March 2022 to 10 July 2022. The full moon and new moon were categorized as distinct experimental groups, with all other dates serving as the control group. Using the direct weighing method, we gathered reliable data on food waste from a total of 1903 tables. Our study examined the impact of the new moon and full moon on food waste while employing propensity score matching (PSM) to control for variables such as dining dates, number of diners, meal timing, spending levels, and restaurant events. The experiment aims to contribute various perspectives to research on food waste, enhance understanding of the cyclic nature of food waste, and provide empirical evidence for the formulation of anti-food-waste policies.

The organization of this paper is as follows. The next section will introduce the selection of experimental dishes, the selection of waste measurement methods, the experimental process, propensity score matching, and data sources. The third part reports the experimental results, specifically including special lunar phases, new moon and full moon, and their impact on food waste. Robustness is discussed in Section 4, while Section 5 includes a brief conclusion.

## 2. Experimental Design and Data Sources

### 2.1. Experimental Design

**Figure 1:**
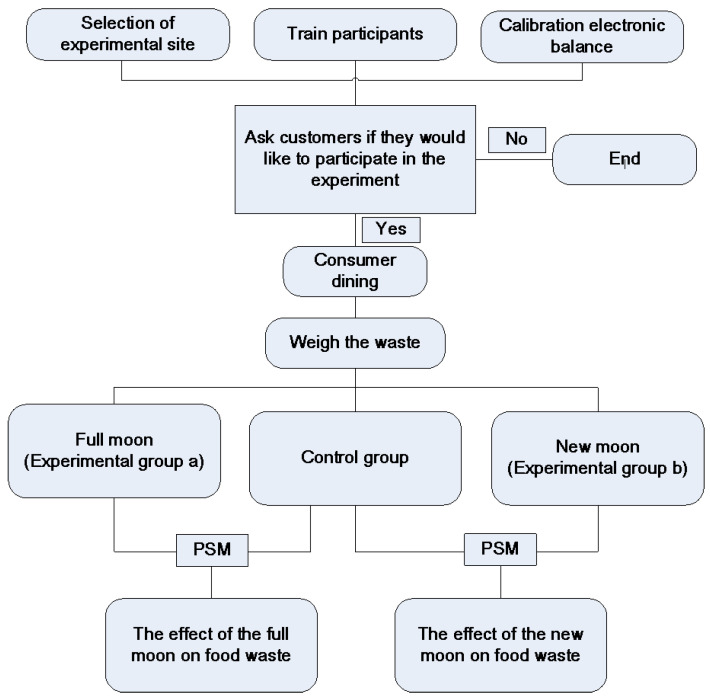
Experiment procedure.

Figure 1 depicts the experimental procedure. Prior to commencing the experiment, we organized a unified training program for the participants, during which we elucidated the experiment’s objectives, procedures, food categorization methods, guidelines for using the electronic scale, and the standard for quantifying food waste. This was carried out to ensure consistent weight measurements and precise evaluation of food waste. Upon entering the hot pot restaurant, the restaurant staff would ask if the guests were willing to take part in the experiment. If they agreed, the restaurant staff would then proceed to measure the amount of food waste per table upon the completion of the patrons’ meal and then calculate the per capita food waste. Conversely, if patrons opted not to participate, food waste measurements would not be conducted. Finally, employing propensity score matching, insights about the impact of the new moon and full moon on the quantity of food waste were derived.

**Figure 2:**
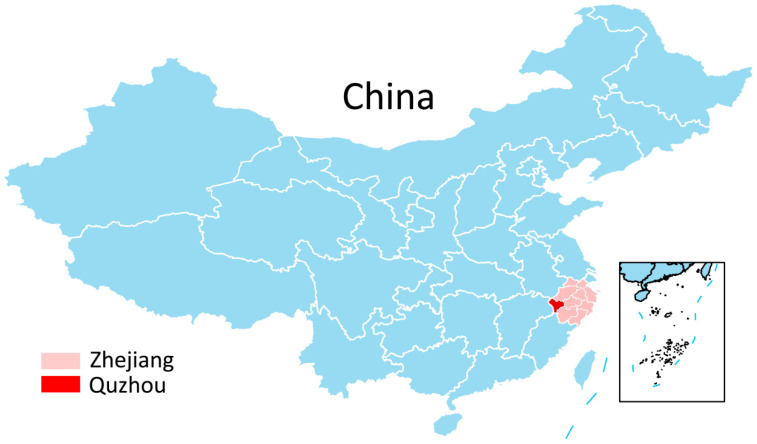
Geographical location of Quzhou.

The experimental site is a hot pot restaurant located in Quzhou City, Zhejiang Province, China (Figure 2). Hot pot is an original culinary delicacy in China, generally referring to the cooking method of boiling various types of food in a special soup stock. According to China Chain-Store and Franchise Association (CCFA) data, hot pot has the highest market share in the Chinese catering industry. There are two main advantages of choosing hot pot as the research subject. First, this cooking method encompasses a comprehensive range of food varieties, facilitating an understanding of the waste situation for each type of food. Second, dishes of the same specifications have fixed weights, making it convenient to obtain information about customers’ ordering quantities.

### 2.2. Propensity Score Matching

Although the experimental procedure strictly adhered to the requirements of a *RCT*, the occurrence frequency of full moons and new moons is relatively low, with only one instance of each in a lunar cycle. To mitigate the potential impact of insufficient sample size on randomness and preserve the credibility of our conclusions, we employed PSM. This technique entailed matching each member of the experimental group with the most comparable member from the control group, thereby minimizing dissimilarities between the two groups in factors unrelated to lunar phases. Previous research [34,35,36] has demonstrated that food waste behavior is influenced by various factors, including dining dates, the number of diners, dining times, spending levels, and store activities. Therefore, based on existing research, we introduced these five influencing factors as matching variables to minimize the impact of other factors on the experiment. Ultimately, we obtained the effects of full moons and new moons on food waste.

(1) $$ATT_{waste}^{full-moon}=Y_{full}-Y_{con}^{PSM-full}$$

(2) $$ATT_{waste}^{new-moon}=Y_{new}-Y_{con}^{PSM-new}$$

When studying the impact of a full moon on food waste, the specific procedure involves considering consumers who dine during a full moon as the treatment group. The average food waste per person in the treatment group is denoted as $Y_{full}$. To establish the control group, PSM is applied to dates excluding the full moon and new moon and takes into account five factors: dining mood, number of diners, dining time, spending level, and store promotions. The average food waste per person in the control group is denoted as $Y_{con}^{PSM-full}$. By calculating the disparity between the treatment and control groups, $ATT_{waste}^{full-moon}$ is ascertained, signifying the impact of a full moon on food waste. Likewise, when assessing the influence of a new moon on food waste, the new moon is considered the treatment group, leading to the evaluation of the impact of a new moon on food waste, denoted as $ATT_{waste}^{new-moon}$.

The propensity score matching estimation starts with a balanced matching test to ensure the reliability of the matching effect. Five variables were selected for matching: dining dates, the number of diners, dining times, spending levels, and store promotions. The matching method was one-to-five matching within a common range. The dining date is a dummy variable, with statutory holidays designated as 0 and workdays as 1. We consider children (under 6 years old) as 0.5 unit when calculating the number of diners. The dining time refers to the period from when the consumer is seated to when the bill is paid. Consumer spending is measured by the total amount spent per table. Store promotions indicate that the store may implement different promotional activities at different times. During the experimental period, the store had a total of three different types of promotional activities, represented by two dummy variables. By employing the PSM technique, the objective is to mitigate the influence of factors beyond lunar phases on both the experimental and control groups.

### 2.3. Data Sources

The study was carried out between 31 March 2022 and 10 July 2022, incorporating three days of full moon and three days of new moon (on April 2nd, June 4th, 5th, and 6th, the hot pot restaurant was closed due to force majeure). Following the exclusion of extreme values, which represented the upper 1% of the data, a total of 1903 valid samples were collected. The proportion of male participants in the study is 42.6%, while female participants make up 57.4%. Children below the age of six constitute 10.3% of the participants, and individuals aged 60 and above account for a mere 0.2% of the sample. The study included 103 varieties of food, which were classified in accordance with the “Food Classification System” outlined in the FAO, as well as relevant literature [37,38]. These food items were sorted into five main categories: animal protein (such as beef, lamb, pork, poultry, and eggs), plant protein (including legumes and soy products), aquatic products, starch (encompassing rice, flour, coarse grains, sweet potatoes, and potatoes), and vegetables (encompassing root vegetables, stem vegetables, leafy vegetables, flower vegetables, fruit vegetables, fungi, and algae). Additionally, we did not include the waste of fruits in the total food wastage, as the fruits in the hot pot restaurant are usually provided in a self-service manner for free and different varieties are offered in different months. To ensure comparability between the experimental group and the control group, the food waste in this study does not encompass fruits. 

The data on food waste were derived through direct weighing. We employed electronic scales with a precision to the gram and a maximum capacity of 5 kg for this purpose, and all scales were calibrated before the commencement of the study. The dishes provided by the hot pot restaurant had a fixed weight and each dish was individually weighed before serving to ensure consistent weight.

(3) $$Waste_{i}=\sum_{j=1}^{11}Raw_{ij}+\sum_{j=1}^{11}Cooked_{ij}/\beta_{j}$$

The average per capita food waste among consumers comprises two components: $Raw_{ij}$ and $Cooked_{ij}/\beta_{j}$. The raw food ($Raw_{ij}$) waste contributes directly to the overall waste, while cooked food waste is recalibrated to its corresponding raw weight ($Cooked_{ij}/\beta_{j}$) through the application of the raw-to-cooked food conversion factor ($\beta_{j}$), prior to its inclusion in the total food waste ($Waste_{i}$). This study focuses specifically on edible waste, referring to the consumable portion of food that remains uneaten. Non-edible components such as bones and food packaging are excluded from the calculation of food waste. Additionally, it should be noted that the hotpot restaurant offers complimentary takeout services and the unfinished food packed for takeout is also excluded from food waste calculations. Descriptive statistics (Table 1) for the variables are as follows:

## 3. Experimental Results

### 3.1. The Impact of the New Moon on Food Waste

**Figure 3:**
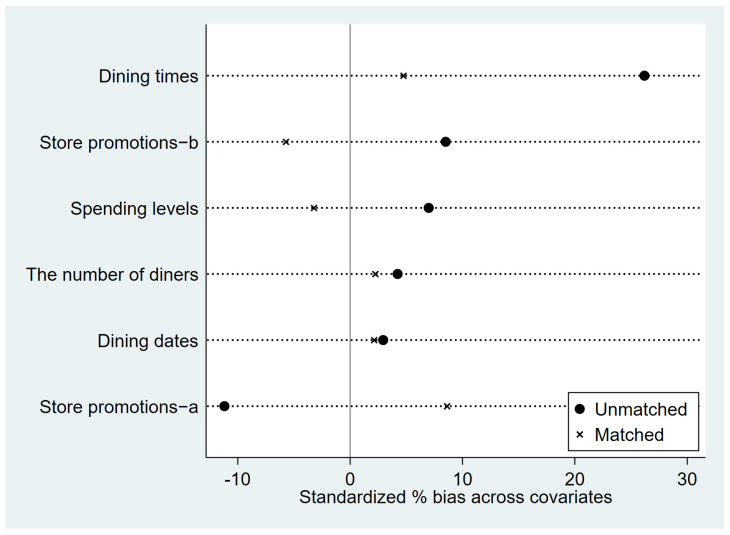
Matching results of PSM for the new moon.

Figure 3 illustrates the matching outcomes of PSM. The circle symbols represent the percentage deviation between the experimental and control groups before matching, while the cross symbols indicate the percentage deviation between the experimental and control groups after matching. It is evident that, prior to matching, the bias percentages of the variables dining times and store promotions-a exceed 10%, indicating significant differences between the experimental and control groups on several variables. Therefore, it is necessary to employ PSM to rectify such discrepancies. After matching, the bias percentages of all variables are below 10%, as shown in Figure 3. Furthermore, each variable’s deviation is closer to the zero axis compared to before PSM, indicating that the post-matching results are superior to the pre-matching outcomes. Employing PSM can significantly minimize the influence of other factors on the experiment and better facilitate the identification of the impact of the new moon on food waste.

**Figure 4:**
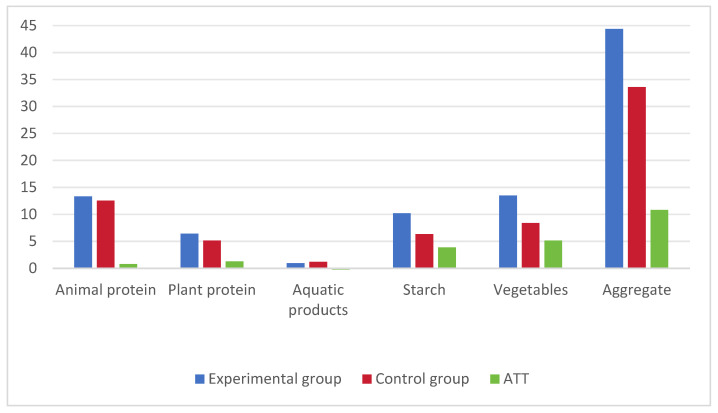
Waste amounts of each food category during the new moon. Note: the control group was obtained by applying PSM to match it with the experimental group (new moon).

Figure 4 depicts the per capita food waste during the new moon phase and in the control group. The blue bars represent the per capita wastage in the experimental group, while the red bars represent the per capita wastage in the control group. The green bars indicate the difference between the experimental and control groups, reflecting the impact of the new moon on food waste. The research findings reveal that consumers waste more food during the new moon phase than at other times. Specifically, there is an average increase of 6.27% in animal protein waste (0.79 g), 24.5% in plant protein waste (1.26 g), 60.95% in starch food waste (3.86 g), and 61.09% in vegetable waste (5.12 g), resulting in a total additional food waste of 32.14% (10.79 g).

### 3.2. The Impact of the Full Moon on Food Waste

**Figure 5:**
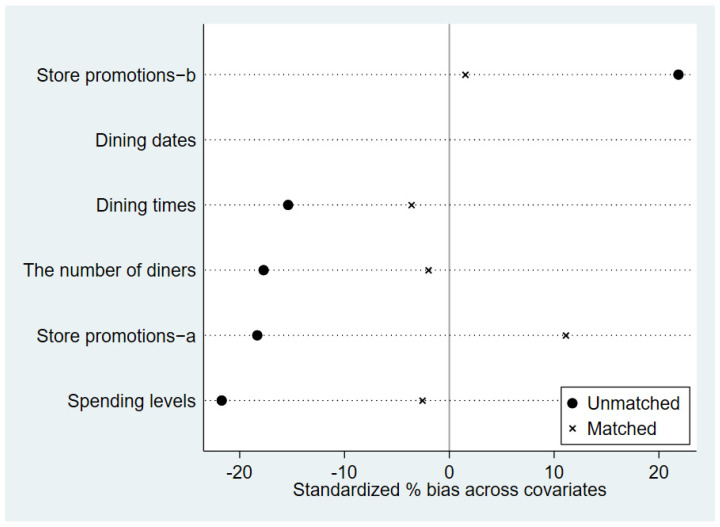
Matching results of PSM for the full moon.

Figure 5 illustrates the outcomes of PSM. Circle symbols represent the percentage deviation between the experimental and control groups prior to the matching process, while cross symbols indicate the percentage deviation after matching. Prior to matching, the deviation percentages of all variables exceeded 10%, indicating significant disparities between the experimental and control groups across multiple factors. After matching, the deviation percentages of variables such as store activities-b, dining times, the number of diners, and spending levels were all below 10%. Furthermore, through the matching procedure, the deviation percentages of each variable were effectively minimized, indicating superior outcomes post-matching compared to pre-matching results. Implementing PSM could significantly mitigate the influence of extraneous variables on the experiment, facilitating an improved assessment of the impact of the full moon on food waste.

**Figure 6:**
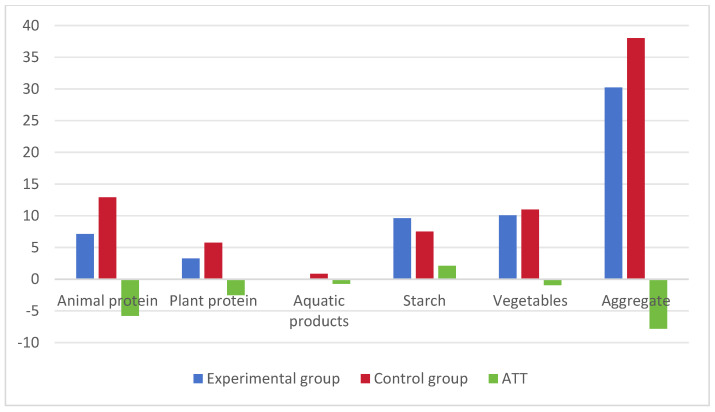
Waste amounts of each food category during the full moon. Note: The control group was obtained by applying PSM to match it with the experimental group (full moon). It should be noted that the conceptual interpretation of the control group in Figure 4 varies from that of the control group in Figure 6.

Figure 6 portrays the per capita food waste during the full moon phase compared to the control group. Similarly, blue represents per capita waste in the experimental group, while red represents per capita waste in the control group, and green indicates the difference between the experimental and control groups, specifically showing the impact of the full moon on food waste. The research shows that consumers waste less food during the full moon period compared to other times. For animal protein, the reduction is 44.65% (5.76 g), for plant protein, it is 43.36% (2.5 g), for aquatic foods, it is 85.39% (0.73 g), and for vegetables, it is 8.43% (0.93 g), resulting in a total reduction of 20.52% (7.80 g) in food waste. During the full moon phase, consumers reduce their food waste by significant amounts. On the other hand, the new moon phase exacerbates consumer food waste, while the full moon phase diminishes it.

### 3.3. Further Research

This section further explores the relationship between lunar phases and food waste, with a primary focus on investigating whether lunar phases influence consumers’ willingness to place orders and subsequently impact food waste. In other words, does the new moon increase consumers’ willingness to place orders, leading to food waste? Conversely, does the full moon decrease consumers’ willingness to place orders, resulting in a reduction in food waste?

To investigate this, we will use the PSM method while maintaining consistency in the matching variables. However, this time we will substitute the explanatory variable with consumers’ order quantity to evaluate the impact of lunar phases on the volume of orders placed.

Table 2 has revealed that, during the new moon phase, the experimental group of consumers ordered an average of 561.88 g of food, while the control group ordered 534.94 g. This indicates a 26.93 g increase in food ordering by the experimental group as compared to the control group. Conversely, during the full moon phase, the experimental group ordered an average of 519.45 g, while the control group ordered 531.26 g, resulting in an 11.80 g decrease in food ordering by the experimental group as compared to the control group. In objective terms, the new moon phase was associated with an increase in consumer willingness to order, which in turn contributed to food waste. Conversely, during the full moon phase, there was a decrease in consumer willingness to order, resulting in a reduction in food waste. In percentage terms, there was a 5% increase in food ordering during the new moon phase, resulting in a 32% increase in food waste. Conversely, there was a 2% decrease in food ordering during the full moon phase, leading to a 21% reduction in food waste. It is evident from this analysis that the order quantity plays a crucial role in determining the occurrence of food waste.

Additionally, during the three instances of full moons in the experiment, the moon was obscured by clouds, rendering it invisible to consumers. In this scenario, both the consumer’s ordering volume and food waste output decreased during the full moon. This indicates that the occurrence of food waste is not directly linked to lunar visibility. Instead, the author is inclined to regard the full moon effect as a cyclic and recurring phenomenon of food wastage outlined in this article.

## 4. Robustness Check

The chapter aims to strengthen the robustness and credibility of the experimental conclusions through the implementation of two methods: expanding the sample size and adjusting the matching model.

### 4.1. Sample Size Enlargement

As previously stated, it was observed that the new moon leads to an increase in consumer waste, while the full moon results in a reduction. However, it is important to note that the sample size for both the new moon and full moon was relatively small compared to the overall sample. During the experimental period, three instances of the new moon occurred, resulting in 59 recorded incidences of food waste data (3.1%). Similarly, the full moon also appeared three times, generating 54 recorded instances of food waste data (2.8%). Some scholars argue that a lack of sufficient samples in the experimental group could threaten the robustness of the conclusions. To address this concern and ensure reliability, we have developed an evaluation mechanism that avoids the need to extend the experiment’s duration while effectively increasing the sample size of the experimental group. Specifically, this approach entails defining the day of the new moon and its adjacent days as a comprehensive broad new moon and replicating the experiment accordingly. If it is determined that the broad new moon causes an increase in consumer waste, albeit to a lesser extent than the previous experimental results, this would confirm the robustness of the conclusion. Similarly, by applying the same methodology to define a comprehensive broad full moon, repeating the experiment, and subsequently observing that the broad full moon generates a reduction in consumer waste that is lower than the previous experimental findings, it would further validate the robustness of the conclusion.

Table 3 presents the findings of the examination, wherein the results obtained from an enlarged sample through repeated experimentation were found to be consistent with the expected results. Specifically, the occurrence of a new moon is associated with an escalation in consumer food waste, whereas a full moon possesses the potential to curtail such waste. Moreover, the PSM demonstrates favorable matching effectiveness even in scenarios with limited sample sizes. By utilizing the method of re-sampling, PSM provides a way to meet the randomization requirements of RCTs more effectively.

### 4.2. Modifying the Matching Model

The current research employed PSM with a nearest neighbor matching (k = 5) to match each participant in the treatment group with the five most similar individuals from the control group. This allowed for the determination of the impact of lunar phase disparities on food waste. To mitigate the possibility of coincidental results stemming from k = 5, the matching method was kept constant while repeating the experiment with k = 3, 4, 6, and 7. In addition to nearest neighbor matching, PSM offers various other matching methods. To ensure the conclusion that gender differences significantly contribute to food waste is robust, the study repeated experiments with various matching methods without altering the matching variables. This was carried out to assess the consistency of the findings and to avoid any specific effect generated by a particular matching method.

Table 4 presents the matching outcomes of nearest neighbor matching, radius matching, kernel matching, and local linear regression matching. Regarding the new moon, the ATT values obtained from nearest neighbor matching (k = 5, 3, 4, 6, 7), radius matching, kernel matching, and local linear regression matching are 10.79 g, 7.61 g, 10.41 g, 9.13 g, 9.24 g, 8.00 g, 6.24 g, and 7.75 g, respectively. These figures demonstrate similarity and show a considerable distance from the null value, indicating an increase in consumer food waste during the new moon. Similarly, for the full moon, the ATT values obtained from nearest neighbor matching (k = 5, 3, 4, 6, 7), radius matching, kernel matching, and local linear regression matching are −7.80 g, −8.00 g, −7.91 g, −8.40 g, −8.95 g, −8.12 g, 5.64 g, and −8.61 g. All the ATT values are negative and significantly deviate from the null value, indicating a reduction in consumer food waste during the full moon. The conclusion remains robust even after changing the model and adjusting the value of k, indicating that the findings are not exclusive to a specific matching method or a particular value of k. It is important to note that the new moon increases consumer food waste, while the full moon reduces it.

In summary, this study demonstrates the robustness and credibility of its conclusions through the enlargement of the sample size and modification of the matching mode.

## 5. Conclusions

Based on a RCT, we have discovered a close relationship between lunar phases and food waste. The experiment, conducted from 31 March 2022 to 10 July 2022, employed the direct weighing method to collect 1903 valid datasets on food waste. Through PSM, we controlled for other influencing factors in the experiment. Research reveals a significant association between lunar phases and food waste. During the new moon phase, consumers increased their ordering quantity, resulting in an increase in food waste. Specifically, per capita waste of animal protein (0.79 g) increased by 6.27%, vegetable protein (1.26 g) increased by 24.5%, starchy foods (3.86 g) increased by 60.95%, and vegetables (5.12 g) increased by 61.09%. In total, food waste increased by 32.14% (10.79 g). Conversely, during the full moon phase, consumers decreased their ordering quantity and subsequently reduced food waste. Specifically, per capita waste of animal protein (5.76 g) decreased by 44.65%, vegetable protein (2.5 g) decreased by 43.36%, seafood (0.73 g) decreased by 85.39%, and vegetables (0.93 g) decreased by 8.43%. In total, food waste decreased by 20.52% (7.80 g). Additionally, it has been observed that even a slight 2% decrease in customer food orders leads to a significant 20.52% reduction in food waste. Therefore, we recommend that customers exercise moderation when placing orders to avoid excessive food waste. It is advised to be particularly mindful of the amount ordered during the new moon period. It is evident from this analysis that the volume of orders plays a pivotal role in influencing the incidence of food waste. Moreover, through sample size enlargement and modification of the matching mode, we have proven the robustness of our experimental conclusions. The research aims to provide new insights into food waste research and enhance public awareness of its cyclical nature. This will provide empirical evidence to guide the development of pertinent anti-food-waste policies.

There are some limitations in this article. First, due to budget constraints and other factors, the sample size is limited to participants from the city of Quzhou in Zhejiang, China. Further investigation is required to determine whether the experimental conclusions can be extrapolated to countries that do not use the lunar calendar. The effect of lunar phases on food waste, specifically whether the new moon and full moon influence customers’ dining choices, remains undetermined, such as their preference for home cooking or ordering takeout. If the new moon and full moon do influence customers’ dining choices, this experiment may suffer from selection bias. Finally, due to the complexity of the lunar phases’ impact on people’s dietary habits, the experiment only discovered a correlation between lunar phases and changes in ordering quantity, thus affecting food waste quantity. The reasons behind this relationship were not further investigated. In the future, the author plans to conduct longer-term experiments to delve into the relationship between lunar phases and food waste, and gather food waste data from countries that do not use the lunar calendar in order to investigate the underlying causes of this phenomenon.

## Figures and Tables

**Table 1:** Sample descriptive statistics.

	Complete Sample (*n* = 1903)				New Moon (*n* = 59)				Full Moon (*n* = 54)			
Variable	Mean	S.D.	Min	Max	Mean	S.D.	Min	Max	Mean	S.D.	Min	Max
Animal protein order quantity (g)	256	109.2	0	1038.0	259.1	105.4	109.2	574.5	254.6	108.1	75.3	539.4
Plant protein order quantity (g)	67.3	103.7	0	1122.4	71.7	102.9	0	484.0	60.1	82.1	0	264.0
Aquatic product order quantity (g)	33.5	30.8	0	267.0	35.1	27.7	0	105.9	36.0	33.6	0	127.1
Starch order quantity (g)	69.0	116.8	0	1000.0	78.5	113.9	0	548.6	59.4	99.6	0	500.0
Vegetable order quantity (g)	110.0	80.1	0	555.6	117.5	74.8	0	325.1	109.4	83.1	0	371.5
Total order quantity (g)	535.9	218.5	0	2409.7	561.9	181.7	217	1096.3	519.5	190.6	199.7	993.6
Animal protein waste (g)	13.6	26.5	0	408.5	13.3	24.2	0	108.6	7.1	14.0	0	57.0
Plant protein waste (g)	4.8	15.2	0	182.0	6.4	15.1	0	75.0	3.3	9.6	0	44.0
Aquatic product waste (g)	0.9	5.1	0	73.5	1.0	3.3	0	14.6	0.1	0.7	0	5.1
Starch waste (g)	7.8	20.3	0	203.8	10.2	24.1	0	138.7	9.6	19.7	0	96.0
Vegetable waste (g)	9.5	21.0	0	189.3	13.5	30.3	0	189.3	10.1	22.3	0	104.2
Total waste (g)	36.6	50.1	0	475.5	44.4	51.6	0	201	30.2	46.3	0	196.9
Dining dates	0.6	0.5	0	1	0.7	0.5	0	1	1	0	1	1
The number of diners	2.9	1.2	1	10	3	1.2	2	7	2.7	1.2	1	7.5
Dining times (min)	68.8	19.5	13	228	73.7	19.5	30	156	65.7	16.8	34	109
Spending levels (CNY)	359.9	125.0	110	1168	367.6	100.3	150	581	335.4	99.0	167	709

Note: Food waste and orders are per capita. The dining date is a dummy variable, with statutory holidays designated as 0 and workdays as 1. We consider children (under 6 years old) as 0.5 unit when calculating the number of diners. The dining time refers to the period from when the consumer is seated to when the bill is paid. Consumer spending is measured by the total amount spent per table.

**Table 2:** Effect of lunar phase on meal ordering volumes.

	Variable	Experimental Group	Control Group	ATT
New moon	Consumption	561.88 g	534.94 g	26.93 g
	Order quantity	44.36 g	33.57 g	10.79 g
Full moon	Consumption	519.45 g	531.26 g	−11.80 g
	Order quantity	30.23 g	38.03 g	−7.80 g

**Table 3:** Results of sample size enlargement.

	Experimental Group	Control Group	ATT
New moon	44.36 g	33.57 g	10.79 g
Broad new moon	41.02 g	35.51 g	5.51 g
Full moon	30.23 g	38.03 g	−7.80 g
Broad full moon	34.24 g	37.28 g	−3.04 g

**Table 4:** Results of different matching methods.

	New Moon			Full Moon		
	Experimental Group	Control Group	ATT	Experimental Group	Control Group	ATT
Nearest neighbor matching (k = 5)	44.36 g	33.57 g	10.79 g	30.23 g	38.03 g	−7.80 g
Nearest neighbor matching (k = 3)	44.36 g	36.75 g	7.61 g	30.23 g	38.23 g	−8.00 g
Nearest neighbor matching (k = 4)	44.36 g	33.95 g	10.41 g	30.23 g	38.14 g	−7.91 g
Nearest neighbor matching (k = 6)	44.36 g	35.23 g	9.13 g	30.23 g	38.63 g	−8.40 g
Nearest neighbor matching (k = 7)	44.36 g	35.12 g	9.24 g	30.23 g	39.18 g	−8.95 g
Radius matching (caliper = 0.05)	44.36 g	36.36 g	8.00 g	30.23 g	38.35 g	−8.12 g
Kernel matching (epan kernel, bandwidth = 0.05)	44.36 g	38.12 g	6.24 g	30.23 g	35.87 g	−5.64 g
Local linear regression matching	44.36 g	36.61 g	7.75 g	30.23 g	38.84 g	−8.61 g

## Data Availability

The original contributions presented in the study are included in the article, further inquiries can be directed to the corresponding author.
